# COVID-19-Induced Hypertriglyceridemia Leading to Pancreatitis in a 51-Year-Old Female

**DOI:** 10.7759/cureus.64065

**Published:** 2024-07-08

**Authors:** Parisa Aijaz, Ji Yoon Park, Harshani Yarlagadda

**Affiliations:** 1 Internal Medicine, Charleston Area Medical Center, Charleston, USA

**Keywords:** covid-19 (corona-virus disease), covid-19 and pancreatitis, clinical case report, hypertriglyceridemia induced pancreatitis, covid-19

## Abstract

There are increasing reports of the effects of COVID-19 on the pancreas. Pancreatitis, as a result of hypertriglyceridemia, has also been reported. Hypothesized mechanisms include hemophagocytic lymphohistiocytosis (HLH) syndrome and acquired lipoprotein lipase (LPL) inhibitors. We present a 51-year-old female patient who presented with nausea, vomiting, and epigastric abdominal pain radiating to the back. On examination, she had generalized abdominal tenderness without guarding or rebound tenderness. Our workup revealed elevated lipase of 1150 units/L, triglycerides (TG) of 11340 mg/dL, and mild pancreatitis on an abdominal computed tomography (CT) scan. On day 2, she developed a new oxygen requirement and tested positive for COVID-19. She was treated with fluids and opiates for pancreatitis, plasmapheresis, and an insulin infusion to treat her hypertriglyceridemia. She was treated with remdesivir for an acute COVID-19 infection. Triglycerides decreased to <500 mg/dL with treatment, and she was discharged home on oral lipid-lowering agents. By discussing this case, we aim to shed light on the association between COVID-19 and hypertriglyceridemia, which can further lead to life-threatening complications such as acute pancreatitis. Further studies are needed to identify the exact mechanisms, preventive measures, and long-term effects of COVID-19 on triglycerides and the pancreas.

## Introduction

The COVID-19 virus was initially noted to mainly affect the lungs, but many other systems are involved. The effect of the COVID-19 virus on the pancreas has been well documented, with many reports of pancreatitis, with a prevalence of 0.27-3.1% reported in recent studies. The pathophysiology involves direct tissue infection, ischemia, or multi-organ dysfunction [1]. There have also been reports of COVID-19-induced hypertriglyceridemia, which in turn causes pancreatitis [2-4]. The mechanisms of COVID-19-induced increased triglyceride (TG) levels are not fully understood but are hypothesized to be related to hemophagocytic lymphohistiocytosis (HLH) syndrome, acquired lipoprotein lipase (LPL) inhibitors, liver failure, or as a side effect of medications [3]. Thus far, the effects are thought to be transient, with TG levels reportedly returning to normal within six months. We present a 51-year-old female with baseline moderately increased TG levels who had an acute rise in TG levels to 11,340 mg/dL during an acute COVID-19 infection.

## Case presentation

**Figure 1:**
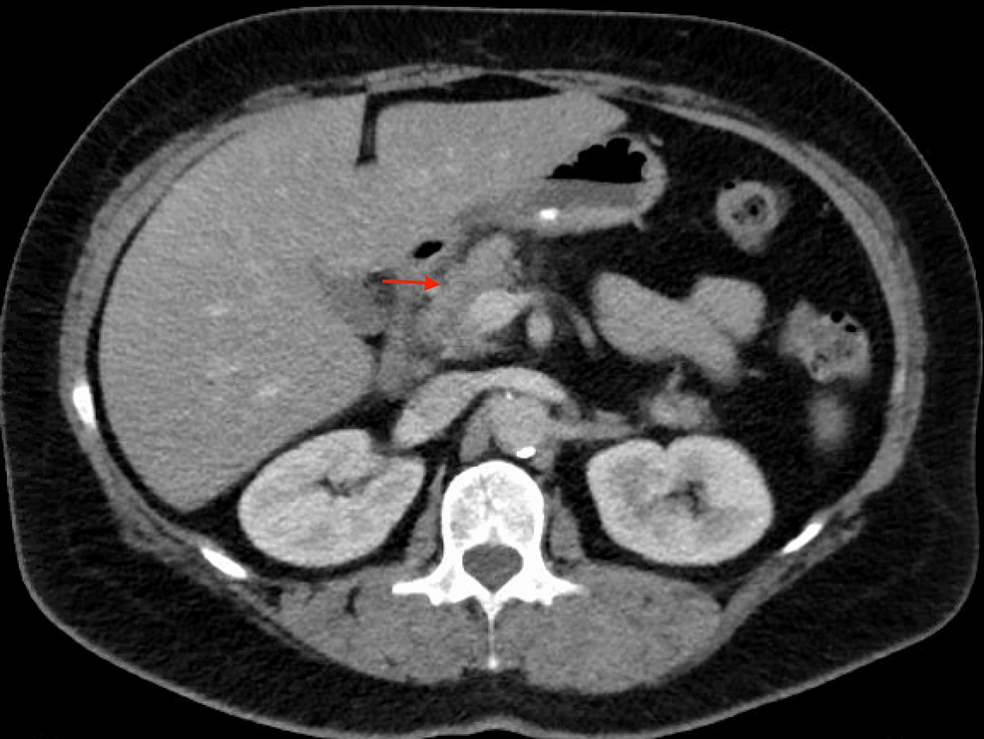
CT of the abdomen of a 51-year-old female with hypertriglyceridemia-induced pancreatitis showing ill-definition at the surface of the pancreatic head that is concerning for acute pancreatitis (red arrow)

A 51-year-old perimenopausal female presented to the emergency department due to sudden severe epigastric pain that radiated to her back, nausea, and vomiting. The pain started while she was having dinner and persistently worsened over the next few hours. She denied current alcohol use, a history of gallstones, or a prior history of pancreatitis. She reported recently elevated triglyceride levels of 760 mg/dL (normal: <150 mg/dL) three months before presentation; however, she was not on any lipid-lowering agents. She had generalized abdominal tenderness on examination without guarding or rebound tenderness. Her initial workup revealed an elevated WBC count, normal hematocrit, normal calcium of 8.7 mg/dL (normal: 8.6-10.3 mg/dL), and elevated lipase of 1150 units/L (normal: 11-82 units/L). Computed tomography (CT) of the abdomen showed an ill-defined border of the pancreatic head, which was concerning for mild pancreatitis (Figure 1). We diagnosed her with acute pancreatitis and initiated treatment with IV fluids and pain medication. The lipid panel reported a triglyceride level of 11,340 mg/dL. We transferred the patient to the intensive care unit. Given her initial mild presentation, a decision was made to initiate an insulin drip at a fixed rate of 0.1 unit/kg/hour with 5% dextrose ringer’s lactate to prevent hypoglycemia. Upon further questioning, she reported the use of oral estrogen for perimenopausal symptoms for the last two years and hydrochlorothiazide for generalized swelling. She denied sudden weight loss, changes in diet, a personal history of coronary artery disease, or a family history of hypertriglyceridemia. Over the next 12 hours, her TGs decreased to 5600 mg/dL with insulin. She initially remained stable, but her condition acutely worsened with abdominal pain, lactic acidosis, an increase in blood urea nitrogen (BUN), and hematocrit. She remained hemodynamically stable. Due to her clinical decline, we discontinued the insulin and proceeded with plasmapheresis (PLEX). After the completion of her first session, her TG further decreased to 1600 mg/dL, and her overall condition improved. On the second day of admission, she complained of shortness of breath and required 2 L of oxygen supplementation. A real-time polymerase chain reaction (RT-PCR) test was positive for COVID-19. The chest X-ray was unremarkable. We treated her with remdesivir (200 mg IV on day 1, followed by 100 mg IV daily for four days). We completed two sessions of plasmapheresis with an insulin infusion between sessions, after which her TG levels decreased below 500 mg/dL. She was then transitioned to oral fenofibrate and icosapent ethyl. Next-generation gene sequencing was negative for genetic mutations pertinent to familial hypertriglyceridemia and familial hypercholesterolemia syndromes. This included mutations in APOA5, APOC2, APOE, CREB3L3, GPD1, GPIHBP1, LCAT, LIPA, LIPC, LMF1, LPL, LRP6, ABCG5, ABCG8, APOB, CETP, CYP27A1, LDLR, LDLRAP1, and PCSK9. Even though her obesity, use of oral estrogen, and thiazides were risk factors for hypertriglyceridemia, she had these risk factors for two years, during which her triglycerides were moderately elevated. After reviewing the current literature, we concluded that her acute increase in TGs was likely secondary to her COVID-19 infection. She eventually made a full recovery and was discharged home on oral medications. Her TGs on discharge were 373 mg/dL (Table 1).

**Table 1:** Progressive change in serum triglyceride levels in response to treatment during the clinical coarse of a patient with hypertriglyceridemia-induced pancreatitis

Intervention	Change in serum triglyceride (TG) levels
None	11,340 mg/dL
Insulin infusion	TG decreased to 5600 mg/dL
First session of plasmapheresis	TG decreased to 1600 mg/dL
Insulin infusion	TG decreased to 1439 mg/dL
Second session of plasmapheresis	TG decreased to <500 mg/dL
Oral fenofibrate and icosapent ethyl	TG remained <500 mg/dL

## Discussion

Identifying and addressing the underlying cause of acute pancreatitis is essential for preventing recurrent episodes. The most common etiologies of acute pancreatitis are gallstones and alcohol consumption [5]. Hypertriglyceridemia is the third leading cause of acute pancreatitis, accounting for approximately 5-25% of episodes [6]. The higher the level of triglycerides, the higher the risk of acute pancreatitis. The prevalence is about 20% if the triglyceride levels are greater than 2,000 mg/dL [6]. The pathway of triglycerides causing pancreatitis is not well understood but has only been hypothesized thus far. The mechanism is thought to occur between the pancreatic lipase and triglyceride-rich lipoprotein interactions within the pancreatic capillaries, which leads to the breakdown of free fatty acids and lysophosphatidylcholine, causing pancreatic damage [6]. The stasis and hypoxicity within the pancreas caused by the lipid breakdown products can further damage the pancreas. The excess free fatty acids can cause the conversion of trypsinogen into trypsin within the acinar cells, causing acinar cell injury [7]. Apart from familial syndromes, some medications can contribute to elevated triglycerides, including oral estrogens and thiazides. We identified hydrochlorothiazide, oral estrogen, and obesity as risk factors in our patients. However, none of these were new in the last two years.

COVID-19 has been associated with hypertriglyceridemia, and the aggregated incidence was 32.98% in pooled COVID-19 patients [2]. Among these reported cases, COVID-induced acute pancreatitis reported triglyceride levels ranging from 150 mg/dl to 4245 mg/dL. One of the reported cases showed TG levels greater than 8300 mg/dl [8]. Our patient had the highest reported levels of triglycerides (11,340 mg/dL) triggered by COVID-19.

HLH syndrome, acquired lipoprotein lipase inhibitors, medication side effects, or acute liver failure are the underlying mechanisms thought to contribute to COVID-19-induced hypertriglyceridemia [3]. HLH causes elevated TG and ferritin levels and reduced fibrinogen levels. HLH is also associated with a cytokine storm, leading to the uncontrolled release of proinflammatory cytokines, particularly IFN-γ, tumor necrosis factor-α (TNF-α), and interleukin-1 (IL-1), IL-2, IL-6, and IL-18 [9]. This leads to dysregulation of lipid metabolism, resulting in impaired clearance of triglycerides and other lipids [pa1] [10]. Less than 5% of adults with severe systemic COVID-19 meet the established HLH criteria, a percentage that may be underestimated due to incomplete information in most reported cases. One study reported that 9.2% of COVID-19 patients with HLH-related features presented with triglyceride concentrations of 150 mg/dL or more [11].

One case study reported acute pancreatitis secondary to severe hypertriglyceridemia due to a transient reduction in lipoprotein lipase (LPL) activity in a patient who recently recovered from COVID-19 [4]. At the time of diagnosis, the patient had markedly reduced LPL activity. Mixing studies with healthy donor plasma demonstrated reduced lipolytic capacity in the donor plasma, indicating an LPL-inhibiting component in the patient’s plasma. They also confirmed reduced LPL quantity using a Western blot. Retesting LPL levels revealed a 20% improvement five months after the initial presentation [4]. The patient was treated with fibrates, which act initially by attaching to peroxisome proliferator-activated receptor alpha (PPAR) receptors, thereby enhancing the activity of LPL [4]. Our institute did not have the resources to test lipoprotein lipase levels in our patients.

The treatment of acute pancreatitis induced by hypertriglyceridemia is similar to other causes of pancreatitis. However, in the setting of hypertriglyceridemia, there is an additional need to lower the triglyceride levels as promptly as possible [6]. Therapies specifically targeting this include insulin infusion, plasmapheresis, heparin infusion, and hemoinfiltration [12]. Insulin therapy has been widely used in treatment. Insulin works by activating lipoprotein lipase (LPL) activity, which can then work to break down chylomicrons to lower triglyceride levels [12]. Along with the initiation of insulin therapy, patients are started on intravenous dextrose 5% to ensure blood glucose levels remain within normal limits. With an insulin infusion, the triglyceride levels are lowered by 50-75% over the next two to three days [12]. The triglyceride levels have typically been reduced to less than 500 mg/dL by three days on average. Another treatment modality is PLEX, which works to rapidly remove triglycerides and chylomicrons from the blood, resulting in decreased inflammation and damage to the pancreas [12]. One session of PLEX can reduce triglyceride levels by up to 50-80% [12]. There are additional risk factors with PLEX when compared to the conservative insulin infusion, including the need for venous access and increased risk for infection, hemorrhage, or thrombosis [12]. There is also a risk of plasma infusion allergic reactions [12]. Insulin infusion is a cost-effective and safe therapy for initiation, with comparable results to PLEX [7].

## Conclusions

Acute pancreatitis is a potentially life-threatening condition with numerous recognized causes. It is crucial to recognize the association between COVID-19 and severe hypertriglyceridemia, as well as its harmful effects on the pancreas. Limited research has been conducted on the underlying pathophysiological processes and long-term outcomes of this association. Further studies are needed to better understand the mechanisms by which COVID-19 induces hypertriglyceridemia, identify patient characteristics that increase the risk, and assess the long-term effects on triglyceride levels and the risk of future pancreatitis episodes.
